# Fast convergence of generalized DeTemple sequences and the relation to the Riemann zeta function

**DOI:** 10.1186/s13660-017-1385-0

**Published:** 2017-05-12

**Authors:** Shanhe Wu, Gabriel Bercu

**Affiliations:** 1 Department of Mathematics, Longyan University, Longyan, Fujian 364012 P.R. China; 2 Department of Mathematics and Computer Sciences, “Dunărea de Jos” University of Galaţi, 111 Domnească Street, Galaţi, 800201 Romania

**Keywords:** 41A60, 41A25, 57Q55, DeTemple sequence, convergence, Euler-Mascheroni constant, approximation, Riemann zeta function

## Abstract

In this paper, we introduce new sequences, which generalize the celebrated DeTemple sequence, having enhanced speed of convergence. We also give a new representation for Euler’s constant in terms of the Riemann zeta function evaluated at positive odd integers.

## Introduction and preliminaries

As is well known, the celebrated Euler-Mascheroni sequence,

$$\gamma _{n}=1+{1\over 2}+\cdots+{1\over n}-\log n \quad (n\geq1), $$

is convergent to the Euler-Mascheroni constant $\gamma =0.5772156649015328\ldots$ , and the rate of convergence is $n^{-1}$. In order to improve the rate of convergence, in [1], DeTemple modified the argument of the logarithm and showed that the sequence

$$R_{n}=1+{1\over 2}+\cdots+{1\over n}-\log \biggl(n+{1\over 2} \biggr) $$

converges quadratically to *γ*. For more details as regards the approximation of the Euler constant with a very high accuracy, we mention the work of Sweeney [2], Bailey [3] and Alzer and Koumandos [4].

There are many famous unsolved problems associated with the properties of this constant; for example it is not known yet if the Euler constant is irrational. The Euler constant is an unusual constant and it seems unrelated to other known constants. However, there are many areas in which Euler’s constant appears, for example, probability theory, random matrix theory and Riemann hypothesis, etc. Recently, Agarwal *et al.* [5], Wang *et al.* [6–9] and Yang *et al.* [10] showed that the Euler-Mascheroni constant has important applications in the field of special functions. In a sense, its appearance in different mathematical areas can be regarded as possible connections between these subjects.

It is the aim of this article to develop a new direction to accelerate the speed of convergence of the sequence $(R_{n})_{n\geq1}$. For this purpose, we consider the sequence

$$\omega _{n}=1+{1\over 2}+\cdots+{1\over n}+x_{n}- \log(n+b_{n}), $$

where $(x_{n})_{n\geq1}$ and $(b_{n})_{n\geq1}$ are suitable sequences, which are chosen in order to increase the speed of convergence of $(\omega _{n})_{n\geq1}$.

Note that we can write $\omega _{n}$ in the form of

$$\omega _{n}=1+{1\over 2}+\cdots+{1\over n}-\log n+x_{n}+\log{1\over 1+{b_{n}\over n}}. $$

If $\lim_{n\to \infty }x_{n}=x$, with $x\in \mathbb {R}$, and $\lim_{n\to \infty }{b_{n}\over n}=0$, then the sequence $(\omega _{n})_{n\geq1}$ is convergent to $\gamma +x$.

To calculate the rate of convergence, we will utilize the Stolz lemma in the case of ${0\over 0}$.

### Lemma 1.1

*Let* $(u_{n})_{n\geq1}$ *and* $(v_{n})_{n\geq1}$ *be two sequences of real numbers*, *having the following properties*:

(i) $\lim_{n\to \infty }u_{n}=\lim_{n\to \infty }v_{n}=0$;
(ii) *the sequence* $(v_{n})_{n\geq1}$ *is strictly decreasing*;
(iii) *there exists* $\lim_{n\to \infty }{u_{n}-u_{n-1}\over v_{n}-v_{n-1}}=\ell$, *with* $\ell\in\mathbb{R}$.

*Then the sequence* $({u_{n}\over v_{n}} )_{n\geq1}$ *is convergent*, *and* $\lim_{n\to \infty }{u_{n}\over v_{n}}=\ell$.

In the following, we are concerned with finding non-vanishing limits of the form

$$\ell=\lim_{n\to \infty }n^{k}(\omega _{n}-\omega )=\lim _{n\to \infty }\frac{\omega _{n}-\omega }{\frac{1}{n^{k}}}, $$

where $\omega =\lim_{n\to \infty }\omega _{n}$. The result will be better as the natural number *k* is large.

Note that

$$\lim_{n\rightarrow\infty}\frac{{\frac{1}{n^{k+1}}}}{{\frac {1}{n^{k}}}-{\frac{1}{(n-1)^{k}}}}=-\frac{1}{k}, $$

using Lemma 1.1, it is enough to find the following limit:

$$\ell=\lim_{n\to \infty }{\omega _{n}-\omega _{n-1}\over {1\over n^{k}}-{1\over (n-1)^{k}}}=-{1\over k} \lim_{n\to \infty }n^{k+1}(\omega _{n}- \omega _{n-1}). $$

We shall write the difference $\omega _{n}-\omega _{n-1}$ as a power series of $n^{-1}$.

First, we arrange the difference $\omega _{n}-\omega _{n-1}$ as

$$\begin{aligned} \omega _{n}-\omega _{n-1} =&{1\over n}+x_{n}-x_{n-1}- \log (n+b_{n})+\log(n-1+b_{n-1}) \\ =&{1\over n}+x_{n}-x_{n-1}-\log \biggl[n \biggl(1+{b_{n}\over n} \biggr) \biggr]+\log \biggl[n \biggl(1+ {b_{n-1}-1\over n} \biggr) \biggr] \\ =&{1\over n}+x_{n}-x_{n-1}-\log \biggl(1+ {b_{n}\over n} \biggr)+\log \biggl(1+{b_{n-1}-1\over n} \biggr). \end{aligned}$$

Then, using the power series representation of $\log(1+x)$, we obtain

$$\begin{aligned} \omega _{n}-\omega _{n-1} =&{1\over n}+x_{n}-x_{n-1}- \biggl[{b_{n}\over n}-{1\over 2} \biggl( {b_{n}\over n} \biggr)^{2}+{1\over 3} \biggl( {b_{n}\over n} \biggr)^{3}-{1\over 4} \biggl( {b_{n}\over n} \biggr)^{4}+\cdots \biggr] \\ &{}+ \biggl[{b_{n-1}-1\over n}-{1\over 2} \biggl( {b_{n-1}-1\over n} \biggr)^{2}+{1\over 3} \biggl( {b_{n-1}-1\over n} \biggr)^{3}-{1\over 4} \biggl( {b_{n-1}-1\over n} \biggr)^{4}+\cdots \biggr]. \end{aligned}$$

After some arrangement, we have

$$\begin{aligned} \omega _{n}-\omega _{n-1} =&{1\over n}+x_{n}-x_{n-1}- {b_{n}\over n}+{b_{n-1}-1\over n}+{b_{n}^{2}-(b_{n-1}-1)^{2}\over 2n^{2}}- {b_{n}^{3}-(b_{n-1}-1)^{3}\over 3n^{3}} \\ &{}+{b_{n}^{4}-(b_{n-1}-1)^{4}\over 4n^{4}}+O \biggl({1\over n^{5}} \biggr), \end{aligned}$$

or more convenient

1.1 $$\begin{aligned} \omega _{n}-\omega _{n-1} =&x_{n}-x_{n-1}- {b_{n}-b_{n-1}\over n}+{b_{n}^{2}-(b_{n-1}-1)^{2}\over 2n^{2}} \\ &{}-{b_{n}^{3}-(b_{n-1}-1)^{3}\over 3n^{3}}+{b_{n}^{4}-(b_{n-1}-1)^{4}\over 4n^{4}}+O \biggl( {1\over n^{5}} \biggr). \end{aligned}$$

The above relational expression (1.1) is useful, it will be used to establish our main results in subsequent sections.

## Sequences of DeTemple type and relevant results

Let us consider certain special cases of $x_{n}-x_{n-1}$ and $b_{n}-b_{n-1}$, which will enable us to establish the generalizations of some sequences from DeTemple [1] and Mortici [11].

Case 1. Suppose $x_{n}-x_{n-1}=0$, for all $n\geq2$ and $b_{n}-b_{n-1}=0$, for all $n\geq2$.

In this case the sequences $(x_{n})_{n\geq1}$ and $(b_{n})_{n\geq1}$ are constant; let $x_{n}=c$, for all $n\geq1$ and $b_{n}=b$, for all $n\geq1$.

In (1.1), the coefficient of ${1\over 2n^{2}}$ becomes $b_{n}^{2}-(b_{n-1}-1)^{2}=b^{2}-(b-1)^{2}=2b-1$.

Subcase 1.1. If $b={1\over 2}$, then the coefficient of ${1\over n^{2}}$ vanishes and

$$\ell=\lim_{n\to \infty } n^{2}(\omega _{n}-\omega )=- {1\over 2}\lim_{n\to \infty }n^{3} \biggl[ {1\over 12 n^{3}}+O \biggl({1\over n^{4}} \biggr) \biggr]=- {1\over 24}, $$

therefore the speed of convergence is $n^{-2}$.

Finally, we obtain the sequence

$$\omega _{n}=1+{1\over 2}+\cdots+{1\over n}+c- \log \biggl(n+{1\over 2} \biggr), $$

which for $c=0$ is the celebrated DeTemple sequence [1].

Subcase 1.2. If $b\neq{1\over 2}$, then the term in ${1\over n^{2}}$ is present, therefore the speed of convergence is $n^{-1}$.

Case 2. If $b_{n}-b_{n-1}=0$, for all $n\geq2$, then the sequence $(b_{n})_{n\geq1}$ is constant. Suppose $b_{n}=b$, for all $n\geq1$ and $b\neq{1\over 2}$. Then

$$\omega _{n}-\omega _{n-1}=x_{n}-x_{n-1}+ {2b-1\over 2n^{2}}-{3b^{2}-3b+1\over 3n^{3}}+O \biggl({1\over n^{4}} \biggr). $$

Subcase 2.1. Suppose $x_{n}-x_{n-1}+{2b-1\over 2n^{2}}=0$ for all $n\geq2$. Taking the sum, we obtain

$$x_{n}=x_{1}+{1-2b\over 2} \biggl( {1\over 2^{2}}+{1\over 3^{2}}+\cdots+{1\over n^{2}} \biggr). $$

The sequence $(\omega_{n})$ becomes

$$ \omega _{n} = 1+{1\over 2}+\cdots+{1\over n}+x_{1}+ {1-2b\over 2} \biggl({1\over 2^{2}}+{1\over 3^{2}}+ \cdots+{1\over n^{2}} \biggr)-\log(n+b) $$

or, in a more suitable form,

2.1 $$ \omega _{n} = 1+x_{1}+\sum _{k=2}^{n}{2k+1-2b\over 2k^{2}}-\log(n+b). $$

The speed of convergence of $(\omega _{n})_{n\geq1}$ is $n^{-2}$. Note that the logarithm function in the above expression has definition for $b\geq0$. Therefore, we obtain the following result.

### Theorem 2.1

*If we denote by* $(\omega _{n})_{n\geq1}$ *the sequence* (2.1) *and by* *ω* *its limit*, *then* $(\omega _{n})_{n\geq1}$ *converges quadratic to* *ω*.

For $b=0$, we obtain the sequence defined in Theorem 3.1 of [11].

Subcase 2.2. Suppose

$$x_{n}-x_{n-1}+{2b-1\over 2n^{2}}+{-3b^{2}+3b-1\over 3n^{3}}=0 \quad \text{for all } n\geq2. $$

Summing side by side, we obtain

$$x_{n}=x_{1}+\sum_{k=2}^{n} \biggl({1-2b\over 2}\cdot{1\over k^{2}}+ {3b^{2}-3b+1\over 3}\cdot{1\over k^{3}} \biggr). $$

The sequence $(\omega_{n})$ has the form

2.2 $$ \omega _{n}=1+x_{1}+\sum _{k=2}^{n} \biggl({1\over k}+ {1-2b\over 2}\cdot{1\over k^{2}}+{3b^{2}-3b+1\over 3} \cdot{1\over k^{3}} \biggr)-\log(n+b). $$

The speed of convergence is $n^{-3}$. Note that the logarithm $\log(n+b)$ has definition for $b\geq0$. This leads to the following assertion.

### Theorem 2.2

*If we denote by* $(\omega _{n})_{n\geq1}$ *the sequence* (2.2) *and by* *ω* *its limit*, *then* $(\omega _{n})_{n\geq1}$ *converges cubic to* *ω*.

For $b=0$, we obtain the sequence defined in Theorem 3.2 of [11].

## Generalized DeTemple sequences

In this section we shall establish two generalized DeTemple sequences in terms of the condition $b_{n}+b_{n-1}-1=0$.

Case 1. Suppose

3.1 $$ x_{n}-x_{n-1}-{b_{n}-b_{n-1}\over n}+ {b_{n}^{2}-(b_{n-1}-1)^{2}\over 2n^{2}}=0 \quad (n\geq2). $$

We would like to find the sequence $(b_{n})_{n\geq1}$ such that $b_{n}+b_{n-1}-1=0$ (this vanishes; the nominator $b_{n}^{2}-(b_{n-1}-1)^{2}=0$). It follows that $b_{n}=-b_{n-1}+1$. Therefore $b_{n}={(-1)^{n-1}\cdot(2b_{1}-1)+1\over 2}$, and this satisfies the initial condition $\lim_{n\to \infty }{b_{n}\over n}=0$. On the other hand, one has $b_{n}-b_{n-1}=(-1)^{n-1}(2b_{1}-1)$.

Equation (3.1) becomes

$$x_{n}-x_{n-1}={(-1)^{n-1}\over n} (2b_{1}-1). $$

Taking the sum from 2 to *n* and fixing $x_{1}=2b_{1}-1$, we obtain

$$x_{n}=(2b_{1}-1) \biggl({1\over 1}- {1\over 2}+{1\over 3}-{1\over 4}+\cdots+ {(-1)^{n-1}\over n} \biggr). $$

We remark that $(x_{n})_{n\geq1}$ is the Leibniz’s sequence multiplied by the constant $(2b_{1}-1)$. This sequence is convergent, having the limit

$$\lim_{n\to \infty } x_{n}=(2b_{1}-1) \log2. $$

Therefore, we obtain the sequence

$$\begin{aligned} \omega _{n} =&1+{1\over 2}+\cdots+{1\over n}+(2b_{1}-1) \biggl(1-{1\over 2}+{1\over 3}-{1\over 4}+ \cdots+{(-1)^{n-1}\over n} \biggr) \\ &{}-\log \biggl[n+{(-1)^{n-1}\cdot(2b_{1}-1)+1\over 2} \biggr], \end{aligned}$$

or, in more suitable form,

3.2 $$ \omega _{n}=\sum_{k=1}^{n} {1+(2b_{1}-1)(-1)^{k-1}\over k}-\log \biggl[n+{(-1)^{n-1}\cdot(2b_{1}-1)+1\over 2} \biggr], $$

with speed of convergence $n^{-2}$ and limit $\gamma +(2b_{1}-1)\log2$.

Note that the logarithm function in the above expression has definition for $b_{1}\in(0,1)$. Hence, we have the following result.

### Theorem 3.1

*If we denote by* $(w_{n})_{n\geq1}$ *the sequence* (3.2), *then* $(w_{n})_{n\geq1}$ *converges quadratically to its limit* $\gamma +(2b_{1}-1)\log2$.

It is clear that for $b_{1}={1\over 2}$ we obtain DeTemple sequence.

Case 2. Note that

$$\begin{aligned} \omega _{n}-\omega _{n-1} =&x_{n}-x_{n-1}- {b_{n}-b_{n-1}\over n}-{b_{n}^{3}-(b_{n-1}-1)^{3}\over 3n^{3}} \\ &{}- \cdots-{b_{n}^{2p+1}-(b_{n-1}-1)^{2p+1}\over (2p+1)n^{2p+1}}+O \biggl(\frac{1}{n^{2p+3}} \biggr). \end{aligned}$$

As above, we consider the sequence $b_{n}={(-1)^{n-1}(2b_{1}-1)+1\over 2}$, $b_{1}\in(0,1)$.

We have seen that it satisfies the equality $b_{n}+b_{n-1}-1=0$. Then $b_{n}^{2}-(b_{n-1}-1)^{2}=0$ and, in general,

$$b_{n}^{2k}-(b_{n-1}-1)^{2k}=0, \quad k=1,2,\ldots. $$

Suppose

$$x_{n}-x_{n-1}={b_{n}-b_{n-1}\over n}+{b_{n}^{3}-(b_{n-1}-1)^{3}\over 3n^{3}}+ \cdots +{b_{n}^{2p+1}-(b_{n-1}-1)^{2p+1}\over (2p+1)n^{2p+1}}. $$

From $b_{n}+b_{n-1}-1=0$, one has $b_{n-1}-1=-b_{n}$. Further, it follows that

$$b_{n}^{2k+1}-({b_{n-1}-1})^{2k+1}=b_{n}^{2k+1}-(-b_{n})^{2k+1} =2b_{n}^{2k+1}. $$

On the other hand,

$$\begin{aligned} {b_{n}-b_{n-1}\over n} =& {b_{1}(-1)^{n-1}+{1-(-1)^{n-1}\over 2}-b_{1}(-1)^{n-2}-{1-(-1)^{n-2}\over 2}\over n} \\ =&{(-1)^{n-1}(2b_{1}-1)\over n}. \end{aligned}$$

Hence, we deduce that

$$x_{n}-x_{n-1}={(-1)^{n-1}(2b_{1}-1)\over n}+{2b_{n}^{3}\over 3n^{3}}+ {2b_{n}^{5}\over 5n^{5}}+\cdots+{2b_{n}^{2p+1}\over (2p+1)n^{2p+1}}. $$

Taking the sum for *k* from 2 to *n*, we have

$$x_{n}-x_{1}=\sum_{k=2}^{n} \biggl[{(-1)^{k-1}(2b_{1}-1)\over k}+{2b_{k}^{3}\over 3k^{3}}+\cdots+ {2b_{k}^{2p+1}\over (2p+1)k^{2p+1}} \biggr]. $$

If we fix

$$x_{1}={2b_{1}-1}+{2b_{1}^{3}\over 3}+ {2b_{1}^{5}\over 5}+\cdots+{2b_{1}^{2p+1}\over 2p+1}, $$

then we obtain

$$x_{n}=\sum_{k=1}^{n} \biggl[ {(-1)^{k-1}(2b_{1}-1)\over k}+{2b_{k}^{3}\over 3k^{3}}+\cdots+{2b_{k}^{2p+1}\over (2p+1)k^{2p+1}} \biggr]. $$

Hence,

$$\begin{aligned} \omega _{n} =&1+{1\over 2}+\cdots+{1\over n}+ \sum_{k=1}^{n} \biggl[{(-1)^{k-1}(2b_{1}-1)\over k}+ {2b_{k}^{3}\over 3k^{3}}+\cdots +{2b_{k}^{2p+1}\over (2p+1)k^{2p+1}} \biggr] \\ &{}-\log \biggl[n+{(-1)^{n-1}(2b_{1}-1)+1\over 2} \biggr] \\ =&\sum_{k=1}^{n}{1\over k}+ \sum_{k=1}^{n} \biggl[{(-1)^{k-1}(2b_{1}-1)\over k}+ {2b_{k}^{3}\over 3k^{3}}+\cdots+{2b_{k}^{2p+1}\over (2p+1)k^{2p+1}} \biggr] \\ &{}-\log \biggl[n+{(-1)^{n-1}(2b_{1}-1)+1\over 2} \biggr] \\ =&\sum_{k=1}^{n}\biggl[2\cdot {(-1)^{k-1}(2b_{1}-1)+1\over 2k}+{2\over 3} \biggl({(-1)^{k-1}(2b_{1}-1)+1\over 2k} \biggr)^{3} \\ &{}+\cdots+{2\over 2p+1} \biggl({(-1)^{k-1}(2b_{1}-1)+1\over 2k} \biggr)^{2p+1}\biggr] \\ &{}-\log \biggl[n+{(-1)^{n-1}(2b_{1}-1)+1\over 2} \biggr]. \end{aligned}$$

Putting $a_{k}={(-1)^{k-1}(2b_{1}-1)+1\over 2k}$, then $\lim_{k\to \infty }a_{k}=0$.

Further,

$$ \omega _{n} = 2\sum_{k=1}^{n} \biggl( {a_{k}\over 1}+{a_{k}^{3}\over 3}+\cdots+{a_{k}^{2p+1}\over 2p+1} \biggr)-\log (n+n a_{n} ). $$

Consequently, we get the following result.

### Theorem 3.2

*The sequence*

$$ \omega _{n} = 2\sum_{k=1}^{n} \biggl( {a_{k}\over 1}+{a_{k}^{3}\over 3}+\cdots+{a_{k}^{2p+1}\over 2p+1} \biggr)-\log (n+n a_{n} ) $$

*has speed of convergence* $n^{-2p-2}$, *where* $a_{k}={(-1)^{k-1}(2b_{1}-1)+1\over 2k}$, $b_{1}\in(0,1)$.

It is easy to observe that $a_{2k}={1-b_{1}\over 2k}$ and $a_{2k+1}={b_{1}\over 2k+1}$.

In order to find the limit of the sequence $(\omega _{n})_{n\geq1}$, we note that it is related to the harmonic sequence $\zeta_{n}(s)=\sum_{k=1}^{n}{1\over k^{s}}$.

For $s>1$, the limit of this sequence defined the celebrated Riemann zeta function $\zeta(s)$, which is very important in mathematics. The speed of convergence of this sequence to its limit is described by the double inequality

$${1\over (s-1)(n+1)^{s-1}}< \zeta(s)-\zeta_{n}(s)< {1\over (s-1)n^{s-1}}. $$

Thus the sequence $(\zeta_{n}(s))_{n\geq1}$ converges with speed of convergence $n^{1-s}$.

A direct calculation gives

$$\begin{aligned} \sum_{k=1}^{\infty}{2a_{k}^{2s+1}\over 2s+1} =& {2\over 2s+1} \bigl(a_{1}^{2s+1}+a_{2}^{2s+1}+ \cdots+a_{n}^{2s+1}+\cdots \bigr) \\ =&{2\over 2s+1} \biggl[ \biggl({b_{1}\over 1} \biggr)^{2s+1}+ \biggl({1-b_{1}\over 2} \biggr)^{2s+1}+ \biggl({b_{1}\over 3} \biggr)^{2s+1}+ \biggl( {1-b_{1}\over 4} \biggr)^{2s+1}+\cdots \biggr] \\ =&{2\over 2s+1}\biggl[b_{1}^{2s+1}\cdot \biggl( {1\over 1^{2s+1}}+{1\over 3^{2s+1}}+{1\over 5^{2s+1}}+\cdots \biggr) \\ &{}+(1-b_{1})^{2s+1}\cdot \biggl({1\over 2^{2s+1}}+ {1\over 4^{2s+1}}+{1\over 6^{2s+1}}+\cdots \biggr)\biggr] \\ =&{2\over 2s+1}\biggl[b_{1}^{2s+1} \biggl( {1\over 1^{2s+1}}+{1\over 2^{2s+1}}+{1\over 3^{2s+1}}+ {1\over 4^{2s+1}}+{1\over 5^{2s+1}}+\cdots \biggr) \\ &{}-b_{1}^{2s+1} \biggl({1\over 2^{2s+1}}+ {1\over 4^{2s+1}}+{1\over 6^{2s+1}}+\cdots \biggr) \\ &{}+(1-b_{1})^{2s+1}\cdot{1\over 2^{2s+1}} \biggl( {1\over 1^{2s+1}}+{1\over 2^{2s+1}}+{1\over 3^{2s+1}}+\cdots \biggr)\biggr]. \end{aligned}$$

Hence,

$$\begin{aligned} \sum_{k=1}^{\infty}{2a_{k}^{2s+1}\over 2s+1} =& {2\over 2s+1}\biggl[b_{1}^{2s+1}\cdot \zeta(2s+1)-b_{1}^{2s+1}\cdot{1\over 2^{2s+1}} \zeta(2s+1) \\ &{}+(1-b_{1})^{2s+1}\cdot{1\over 2^{2s+1}}\zeta(2s+1) \biggr] \\ =&{2\over 2s+1}\cdot {b_{1}^{2s+1}(2^{2s+1}-1)+(1-b_{1})^{2s+1}\over 2^{2s+1}}\cdot\zeta (2s+1) \\ =&{b_{1}^{2s+1}(2^{2s+1}-1)+(1-b_{1})^{2s+1}\over (2s+1)\cdot2^{2s}}\cdot\zeta(2s+1), \end{aligned}$$

where $s\in\{1,2,\ldots,p\}$.

We have showed in Case 1 that $\lim_{n\to \infty }\sum_{k=1}^{n}{2a_{k}}-\log (n+na_{n})=\gamma +(2b_{1}-1)\log2$.

Therefore, we obtain the following theorem.

### Theorem 3.3

*The sequence* $(\omega_{n})_{n\geq1}$ *defined by Theorem* 3.2 *converges to*

$$\gamma +(2b_{1}-1)\log2+\sum_{s=1}^{p} {b_{1}^{2s+1}(2^{2s+1}-1)+(1-b_{1})^{2s+1}\over 2^{2s}(2s+1)}\zeta(2s+1) $$

*with speed of convergence* $n^{-2p-2}$.

If we take $b_{1}={1\over 2}$ in Theorem 3.3, then $a_{k}={1\over 2k}$,

3.3 $$\begin{aligned} \omega _{n} =&\sum_{k=1}^{n}2 \biggl[ {1\over 2k}+{1\over 3\cdot2^{3}k^{3}}+\cdots+{1\over (2p+1)2^{2p+1}k^{2p+1}} \biggr] \\ &{}-\log \biggl(n+{1\over 2} \biggr) \\ =&R_{n}+\sum_{k=1}^{n} \biggl[ {1\over 3\cdot2^{2} k^{3}}+\cdots+{1\over (2p+1) 2^{2p} k^{2p+1}} \biggr], \end{aligned}$$

where

$$R_{n}=1+{1\over 2}+\cdots+{1\over n}-\log \biggl(n+{1\over 2} \biggr). $$

Thus, we obtain the following assertion.

### Corollary 3.1

*The sequence* $(\omega _{n})_{n\geq1}$ *defined by* (3.3) *converges to*

$$\gamma +\sum_{s=1}^{p}{1\over (2s+1)2^{2s}} \zeta(2s+1) $$

*with speed of convergence* $n^{-2p-2}$.

Particular cases. For $p=0$, the sequence $(\omega _{n})_{n\geq1}$ is a DeTemple sequence.

For $p=1$, the sequence $(\omega _{n})_{n\geq1}$ is the sequence defined in Theorem 4.1 of [12].

For $p=2$, the sequence $(\omega _{n})_{n\geq1}$ is the sequence defined in Theorem 4.2 of [12].

We remark that the expressions from Theorem 3.3 and Corollary 3.1 concern the expansion of Euler’s constant in terms of the Riemann zeta function evaluated at positive odd integers. Therefore the constant *γ* can be approximated with a very high speed of convergence by the above expansions. For more details of the series representations of the Euler constant, we mention the work of Alzer, Karayannakis and Srivastava [12], Alzer and Koumandos [13] and Sofo [14].

## Conclusion

Our method for accelerating the convergence of DeTemple type sequences is more general than the method introduced in [11] and [15] by Mortici. We support this statement by the large degree of freedom in choosing the sequences $(x_{n})_{n\geq1}$, and $(b_{n})_{n\geq1}$. In this way, we generalized sequences from [11] and introduced two new sequences which generalized DeTemple sequence $(R_{n})_{n\geq1}$ having faster convergence rate.
